# Screening agars for MRSA: evaluation of a stepwise diagnostic approach with two different selective agars for the screening for methicillin-resistant *Staphylococcus aureus* (MRSA)

**DOI:** 10.1186/s40779-015-0046-1

**Published:** 2015-07-21

**Authors:** Volker Micheel, Benedikt Hogan, Thomas Köller, Philipp Warnke, Sabine Crusius, Rebecca Hinz, Ralf Matthias Hagen, Norbert Georg Schwarz, Hagen Frickmann

**Affiliations:** Department of Tropical Medicine at the Bernhard Nocht Institute, German Armed Forces Hospital of Hamburg, Hamburg, Germany; Department of Infectious Disease Epidemiology, Bernhard Nocht Institute for Tropical Medicine Hamburg, Hamburg, Germany; Institute for Microbiology, Virology and Hygiene, University Medicine Rostock, Rostock, Germany

**Keywords:** Methicillin-resistant *Staphylococcus aureus*, Selective agar, Chromogenic agar, Screening, Hygiene, Stepwise diagnostics, CHROMagar, ChromID agar

## Abstract

**Background:**

Colonization with methicillin-resistant *Staphylococcus aureus* (MRSA) poses a hygiene risk that does not spare field hospitals or military medical field camps during military deployments. Diagnostic options for unambiguously identifying MRSA isolates are usually scarce in military environments. In this study, we assessed the stepwise application of two different selective agars for the specific identification of MRSA in screening analyses.

**Methods:**

Nasal swabs from 1541 volunteers were subjected to thioglycollate broth enrichment and subsequently screened on CHROMagar MRSA selective agar for the identification of MRSA. The MRSA identity of suspicious-looking colonies was confirmed afterwards or excluded by another selective agar, chromID MRSA. All isolates from the selective agars with MRSA-specific colony morphology were identified by biochemical methods and mass spectrometry.

**Results:**

The initial CHROMagar MRSA screening identified suspicious colonies in 36 out of 1541 samples. A total of 25 of these 36 isolates showed MRSA-like growth on chromID agar. Out of these 25 isolates, 24 were confirmed as MRSA, while one isolate was identified as *Staphylococcus kloosii*. From the 11 strains that did not show suspicious growth on chromID agar, 3 were methicillin-sensitive *Staphylococcus aureus* (MSSA, with one instance of co-colonization with *Corynebacterium* spp.), 2 were confirmed as MRSA (with 1 instance of co-colonization with MSSA), 2 were lost during passaging and could not be re-cultured, one could not be identified by the applied approaches, and the remaining 3 strains were identified as *Staphylococcus saprophyticus*, *Staphylococcus hominis* (co-colonized with *Macrococcus caseolyticus*) and *Staphylococcus cohnii*, respectively.

**Conclusions:**

The application of the selective agar CHROMagar MRSA alone proved to be too non-specific to allow for a reliable diagnosis of the presence of MRSA. The combined use of two selective agars in a stepwise approach reduced this non-specificity with an acceptably low loss of sensitivity. Accordingly, such a stepwise screening approach might be an option for resource-restricted military medical field camps.

## Background

Colonization with methicillin-resistant *Staphylococcus aureus* (MRSA) poses a relevant public health threat worldwide. Soldiers are at risk as well, both during exercises and on deployment. MRSA is readily transmissible and shows high tenacity, potentially leading to outbreak situations as previously occurred for military trainees in San Diego, California [1]. The nostrils and mucous membranes of the upper respiratory tract are typical sites of colonization [2] from which nosocomial spread may occur. Such colonization with MRSA has been demonstrated for US soldiers irrespective of their current deployment situations [3].

The disease spectrum caused by *Staphylococcus aureus* ranges from mild to moderate skin and soft tissue infections to life-threatening systemic infections [4]. Impressively high percentages of MRSA-induced abscesses have been identified in American soldiers [5].

Increasing numbers of MRSA strains have been isolated in hospitals and community settings since the introduction of beta-lactam antibiotics [6, 7]. Once introduced, community-associated MRSA strains can readily spread within healthcare facilities [8] and pose a considerable risk for immunologically compromised trauma patients within military medial field hospitals or camps on deployment.

Reliable screening procedures are needed for the deployment setting to prevent the spread of MRSA from colonized patients by the enforcement of adequate hygiene precautions. Point-of-care PCR systems such as GeneXpert (Cepheid, Sunnyvale, California) deliver rapid results [9]. However, the specificity of the GeneXpert system has been shown to be reduced in cases of *mecA* gene loss [10, 11]; furthermore, such point-of-care devices are not always available.

Chromogenic selective agars are inexpensive and ready-to-use alternatives or amendments [12] if the basic laboratory infrastructure needed for the growth of bacteria can be provided. Numerous evaluations of MRSA-selective agars have been introduced [13–56] with acceptable but still improvable results regarding their sensitivities and specificities. In this study, we evaluated a stepwise approach using one selective agar as a screening method and another agar for confirmation testing.

## Methods

A total of 1541 nasal swabs for MRSA screening (agar gel transport single plastic swabs without charcoal, Amies w/o Ch, Copan Italia SpA, Brescia, Italy, 108C.USE) were included in the analysis. The nasal vestibulum of healthy adult volunteers was screened using one swab per study participant. The study population comprised Madagascan civilians. To ensure the privacy of the volunteers, no further details regarding the sampling setting are presented for this technical evaluation in accord with our institutes’ ethical standards, which prohibit the assessment and presentation of patient data that do not directly impact the scientific question being assessed. The descriptive analysis of the laboratory procedures presented here does not demand a more detailed presentation of the patient data.

After broth enrichment of the samples in thioglycollate broth (BD, Heidelberg, Germany) for 16–24 h at 37 °C, the cultures were grown on the MRSA selective agar CHROMagar MRSA (MAST Diagnostica, Reinfeld, Germany) for 40–48 h at 37 °C as an initial screening step. Suspicious colonies on the CHROMagar MRSA plates, i.e., mauve-colored colonies, were selected and stored at -80 °C in Microbank tubes (Pro-Lab Diagnostics, Bromborough, UK) until further assessment.

As a next step, all isolated strains were grown on another MRSA-selective chromogenic agar, chromID MRSA (bioMérieux, Marcy-l’Étoile, France, reference: Best.-Nr.43 451 (20 units) or Best.-Nr.43 459 (100 units)), for an additional 40–48 h at 37 °C to confirm or contradict the result of the CHROMagar MRSA-based screening. The isolates were grown directly from the Microbank tubes.

All suspicious isolates that were confirmed by chromID MRSA testing, i.e., green colonies on the chromID MRSA agar, were subjected to matrix-assisted laser-desorption-ionization time-of-flight mass-spectrometry (MALDI-TOF-MS) by a Shimadzu/Kratos “AXIMA Assurance” MALDI-TOF mass spectrometer (SHIMADZU Germany Ltd., Duisburg, Germany) as described [57] for confirmation on the species level. Spectral fingerprints were analyzed using Vitek MS IVD V2 database MS-CE version CLI 2.0.0 (bioMérieux). Commercial BD MAX MRSA PCR (BD, Heidelberg, Germany) was used to verify the identity as MRSA.

Isolates from the CHROMagar MRSA that were not confirmed as MRSA by the growth of suspicious colonies on chromID MRSA were subjected to the following: repeated growth on chromID MRSA agar, penicillin-binding protein 2a (pbp-2a)-latex agglutination (PBP2a Culture Colony Test, Alere Scarborough Inc., Scarborough, Maine, USA), Pastorex Staph-Plus (Bio-Rad, Marnes-la-Coquette, France) latex agglutination targeting clumping factor, protein A and capsular polysaccharides, VITEK-II GP-card (bioMérieux)-based identification and MALDI-TOF-MS (as described above). Antimicrobial susceptibility testing of *Staphylococcus aureus* strains was performed using the VITEK-II AST-P619-card (bioMérieux) to confirm oxacillin resistance.

For MRSA strains that were incorrectly missed by the chromID MRSA agar confirmation testing, *spa* typing was performed using the Ridom StaphType standard protocol for PCR and subsequent double-strand sequencing [58, 59]. Automated sequence allocation was performed using the software Ridom StaphType version 2.2.1 (Ridom Ltd., Würzburg, Germany).

This study was approved by the Ethical Committee of the Ministry of Health of the Republic of Madagascar prior to being conducted (No. 081 - MSANP/CE, 5^th^ November 2012).

## Results and discussion

From 36 out of the analyzed 1541 nasal swabs from healthy volunteers, MRSA-suspicious colonies were isolated from the CHROMagar plates after thioglycollate broth enrichment and freezing at -80 °C in Microbank tubes. As reported from previous studies, the sensitivity and specificity of this selective agar for MRSA ranges from 95.4 % to 100 % and 95 % to 100 %, respectively [60–62]. From these 36 strains, 2 did not grow after freezing, so their identity could not be assessed. It is thus theoretically possible that these strains were indeed MRSA.

**Fig. 1:**
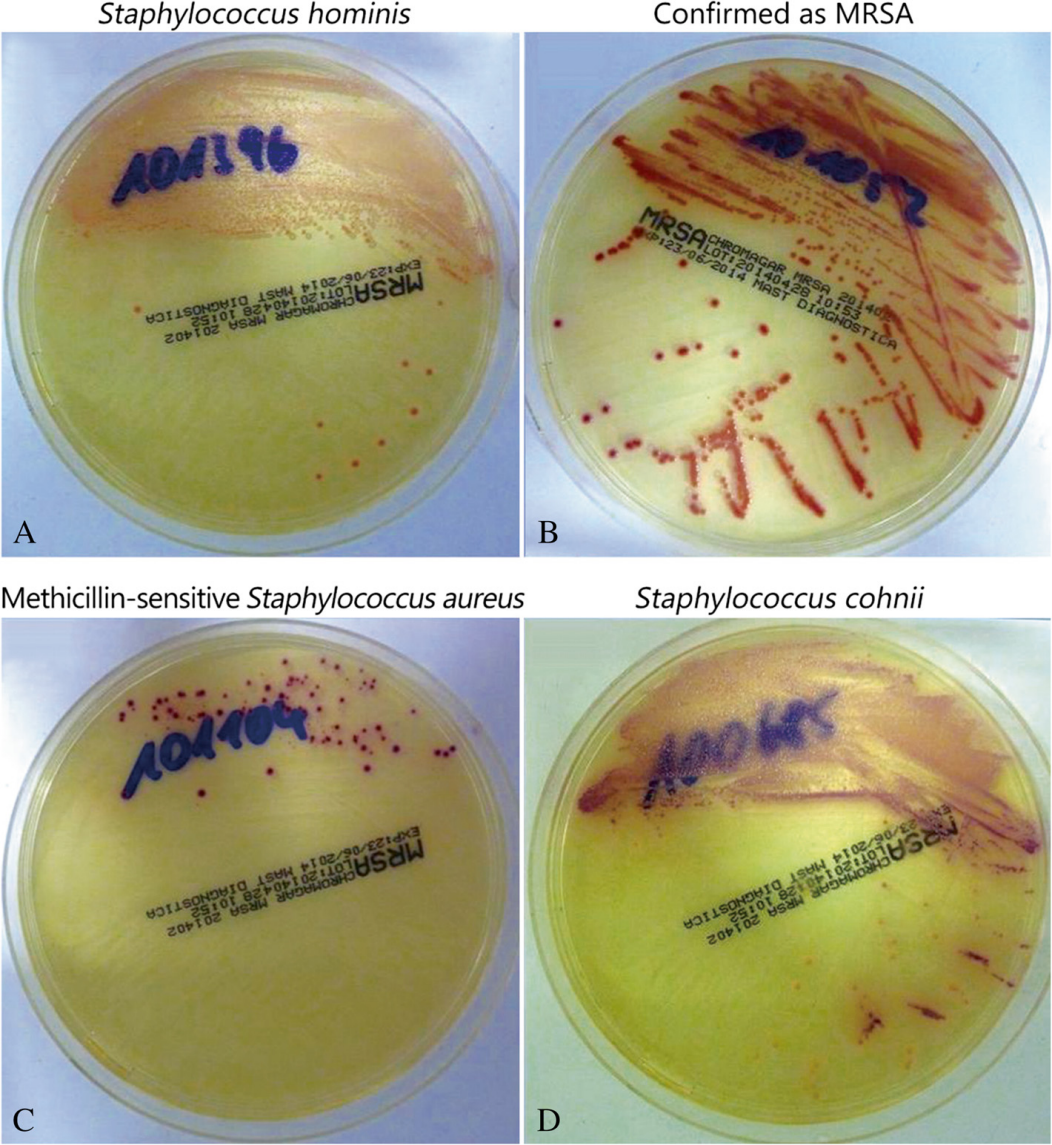
Difficult to discriminate: colony morphology as observed during the CHROMagar MRSA screening. **a**) Staphylococcus hominis. **b**) Confirmed as MRSA. **c**) Methicillin-sensitive Staphylococcus aureus. **d**) Staphylococcus cohnii

For the remaining 34 strains, 25 grew on the chromID MRSA agar as MRSA-like green colonies. The previously described ranges of sensitivity and specificity of chromID MRSA agar are 64.5 % to 99.4 % and 98.5 % to 99.4 %, respectively, for the detection of MRSA [63]. From the 25 strains showing green colonies, 24 out of 25 were confirmed as *Staphylococcus aureus* by MALDI-TOF-MS and as MRSA by the commercial BD MAX MRSA PCR. However, one strain was misidentified by the combined approach based on CHROMagar MRSA and chromID MRSA agar. MALDI-TOF-MS analysis identified this strain as *Staphylococcus kloosii*, a coagulase-negative skin colonizer. For the remaining 9 samples, no growth of the culture or growth of non-suspicious white colonies were observed on chromID MRSA agar. Photographs showing the typical examples of the colony morphology on CHROMagar MRSA for 4 out of these 9 samples are depicted in Fig. 1.

These 9 samples were further investigated by mass spectrometry and biochemical approaches. As described in previous reports [61], small purple colonies of skin-colonizing *Corynebacterium* spp. on CHROMagar MRSA medium might be misidentified as potential MRSA strains by non-experienced investigators.

For one sample, the identification of tiny colonies failed. For three samples, the colonies were identified as coagulase-negative staphylococci, while the VITEK-II GP-cards and MALDI-TOF-MS unanimously identified *Staphylococcus saprophyticus*, *Staphylococcus hominis* and *Staphylococcus cohnii* in these cases. From the sample containing the *Staphylococcus hominis* strain, the skin-colonizer *Macrococcus caseolyticus* was also isolated and identified by mass spectrometry. In another three samples, methicillin-sensitive *Staphylococcus aureus* (MSSA) was confirmed; methicillin-susceptibility was monitored by pbp-2a-agglutination and VITEK-II AST-619 antimicrobial susceptibility testing. *Corynebacterium* spp. was observed to be co-colonized with MSSA in one instance.

MRSA was confirmed in two of nine samples, in spite of an initial non-suspicious growth on chromID MRSA agar. However, after repeated growth on chromID MRSA agar, typical green colonies were identified. One of these two MRSA strains did not show characteristically green colonies earlier than 48 h of growth; an additional MSSA strain was isolated from this same sample. The reasons remain unclear why those two additional MRSA strains did not show typical colony color and morphology during the first assessment on chromID MRSA agar. A possible reason might be the fact that the first growth on chromID MRSA agar was performed directly from the Microbank tubes, as strains can tend to grow atypically immediately after thawing after prolonged storage at -80 °C.

To further characterize the two MRSA strains that were missed by initial chromID MRSA agar confirmation testing, *spa* typing [58, 59] was performed. This rapid and easy-to-use sequence-based molecular tool allows for international phylogenetic comparisons and has widely replaced pulsed field gel electrophoresis as the new gold standard of *Staphylococcus aureus* typing in Germany [64]. The identified *spa* types were t314 for the strain that grew after 48 h of incubation on chromID MRSA agar only and t186 for the other strain. The t314 clone is known to be prevalent in Western Africa [65] and the t186 clone in Eastern Africa [66].

In summary, MRSA strains were identified by the applied procedures in 26 of 1541 samples. A combined approach of CHROMagar MRSA selective agar as a screening tool and chromID MRSA agar as a confirmation tool after thioglycollate broth enrichment identified 24 out of 26 MRSA strains but initially missed two strains. In contrast, a *Staphylococcus kloosii* strain, which is rarely isolated from human samples, was erroneously considered to be MRSA.

However, one incorrectly attributed strain out of a total of 1541 samples represents an acceptable specificity of the approach. The total sensitivity cannot be estimated, as there was no comparative gold standard for the CHROMagar MRSA screening. Accordingly, one cannot say how many MRSA strains were missed by the initial screening.

The significant advantage of CHROMagar MRSA is the fact that the manufacturer sells the agar not only as ready-to-use agar plates but also as a powder, allowing for easy shipment without proper cooling and production of the plates at the site of deployment. However, if the storage of the powder for up to 2 years is intended, the manufacturer recommends storage at 2 °C to 8 °C. The high number of false positive results due to CHROMagar MRSA screening alone is a point of concern, resulting in an insufficient specificity. The design of the study does not allow for statements on the specificity of a screening based on the solitary use of chromID MRSA agar, as this selective agar was used for confirmation testing only.

However, this was not the only limitation of this study. The fact that the initial inoculation was confined to just one MRSA screening agar was admittedly a major drawback. Accordingly, no proper comparison was possible. The stepwise approach described in this study was a description of field experience and not a comparative study.

The consequences of incorrect suspicions of colonization with MRSA are considerable. This is particularly true if isolation of MRSA-positive patients is enforced as a strategy to prevent nosocomial spread in the field hospital. As shown in several studies [67, 68], the isolation of colonized patients leads to reduced nursing quality with poorer outcomes as a result. Accordingly, the usefulness of screening procedures has been discussed with much controversy [69]. The repeated disinfecting washing of all patients without regard to colonization status would be an alternative regarding infection control [70–72] but has not yet been implemented widely.

## Conclusions

If MRSA screening is needed in a resource-restricted medical field camp environment where no alternative approaches for reliable confirmation testing are available, screening with two different MRSA selective agars is an option with an acceptable specificity. CHROMagar MRSA powder can be easily shipped to sites of tropical deployment but bears the risk of misidentifying coagulase-negative staphylococci and MSSA as MRSA if it is used without confirmation testing. Due to the harmful consequences of isolation of MRSA-colonized patients, incorrect identification of colonization with MRSA should be avoided.

## Abbreviations

AST: Antibiotic susceptibility testing
MALDI-TOF-MS: Matrix-assisted laser desorption-ionization time-of-flight mass spectrometry
MRSA: Methicillin-resistant *Staphylococcus aureus*
MSSA: Methicillin-sensitive *Staphylococcus aureus*
PBP: Penicillin binding protein
PCR: Polymerase chain reaction
SPP: Species (plural)
